# Negative Predictive Value of Allergologic Work-Up with Iodinated Contrast Media in ‘Real-Life’ Practice

**DOI:** 10.3390/jcm15145425

**Published:** 2026-07-10

**Authors:** Krzysztof Specjalski, Ilona Iwaszko, Dominik Typiak, Dawid Juszkiewicz, Wiktoria Szablewska, Mateusz Skotnicki, Nicola Le, Michał Powietrzyński, Adam Strukowski, Joanna Śledzik, Marta Chełmińska, Marek Niedoszytko

**Affiliations:** Department of Allergology, Medical University of Gdańsk, ul. Smoluchowskiego 17, 80-952 Gdańsk, Poland; krzysztof.specjalski@gumed.edu.pl (K.S.); dtypiak@gumed.edu.pl (D.T.); dawid.juszkiewicz@gumed.edu.pl (D.J.); w.szablewska@gumed.edu.pl (W.S.); mateuszskotnicki@gumed.edu.pl (M.S.); nicolale@gumed.edu.pl (N.L.); powmichal@gumed.edu.pl (M.P.); adam.strukowski@gumed.edu.pl (A.S.); joannasledzik@gumed.edu.pl (J.Ś.); marta.chelminska@gumed.edu.pl (M.C.); marek.niedoszytko@gumed.edu.pl (M.N.)

**Keywords:** iodinated contrast media, hypersensitivity reactions, drug allergy, drug provocation tests

## Abstract

**Background:** Several strategies such as premedication or allergologic work-up have been used to facilitate safe iodinated contrast media (ICM) application. The aim of this study was to assess the negative predictive value of skin tests and complete allergologic work-up with iodinated contrast media in real-life settings. **Methods:** We retrospectively enrolled 210 patients with a history of immediate hypersensitivity reactions to ICMs. The stepwise protocol of selecting safe ICMs included skin prick tests (SPTs), intracutaneous tests (ICTs), and intravenous drug provocation tests (DPTs). The tests were performed with one or more of the following medications: iohexol, iodixanol, iomeprol, iopromide, and ioversol. At least 6 months after selecting safe ICMs, a telephone follow-up was scheduled. Based on this data, negative predictive values of skin tests and the whole work-up were calculated. **Results:** Overall, we obtained the following rates of positive tests: SPT—12% (55/441); ICT—10% (40/383); and DPT—5% (10/195). The highest rates of positive work-ups were found in patients tested within 1 year after the reaction and patients with a history of urticaria/angioedema after ICMs. The skin tests and DPTs determined a safe ICM in 184/210 (87%) patients. The negative predictive value of skin tests in reference to DPTs in hospital settings was 95%. Finally, a telephone follow-up demonstrated that 75/184 participants (41%) had been given the recommended ICM, including 68 cases (37%) with no subsequent hypersensitivity reaction and 7 (4%) with a reaction. The negative predictive value of allergologic work-up consisting of SPTs, ICTs and DPTs in real-life settings was 90%. **Conclusions:** Skin tests and intravenous drug provocation tests with ICMs are characterised by high negative predictive value, and enable the determination of safe ICMs for most patients.

## 1. Introduction

Iodinated contrast media (ICMs) are widely used in several diagnostic and therapeutic procedures to improve imaging quality. The first attempts at their application were mentioned in the 1920s. However, due to the toxicity and poor performance of the first available ICMs, they were not used in everyday practice until the 1950s. Nowadays, millions of doses of ICMs are administered worldwide each year, mostly during computed tomography (CT) and angiographies [1]. It is estimated that the number of CT examinations is increasing by 4% per year for a total of approximately 300 million CT scans per year, 40% of which are contrast-enhanced [2]. Percutaneous coronary interventions are the most common therapeutic procedures with the use of ICMs. About 4 million coronary angiograms are performed each year in Europe and the United States [3].

All currently used ICMs share a chemical structure based on 2,4,6-tri-iodinated benzene rings [4,5]. Iodine atoms arranged in the benzene rings significantly increase molecule size, causing attenuation of X-rays. There are four major structural classes of ICMs. Their compounds constitute either a single tri-iodinated benzene ring (i.e., monomers) or a pair of tri-iodinated benzene rings linked by an organic functional group (i.e., dimers). Furthermore, the tendency of an ion to form a bond with another ion is governed by the presence (i.e., ionic) or absence (i.e., non-ionic) of a carboxylate functional group on an organic side chain [4]. Ionic monomers are also referred to as ‘high-osmolarity’ agents because they have the weakest ability to attenuate X-rays, and thus need to be administered in high concentrations that are hyperosmolar (have 5–8 times the osmolality of plasma). The remaining classes mentioned are designated as low-osmolar (have 2–3 times the osmolality of serum) [4,5]. The osmolality of these non-ionic dimeric ICMs is comparable to that of normal serum, so they are classified as isotonic or iso-osmotic ICMs [6] (Table 1).

Adverse reactions to ICMs are quite common (up to 15% of patients), but their prevalence largely depends on chemical properties of the molecule. It has been well documented that new agents, characterised by low osmolality, are generally less toxic, and have fewer adverse effects. Hypersensitivity reactions to ICMs are traditionally divided into immediate [7,8], with symptoms developing within 1 h, and non-immediate, which usually occur several hours after ICM administration [9,10,11,12]. They represent a broad spectrum of clinical pictures, from mild, self-limiting skin rashes to severe and life-threatening anaphylactic reactions. Furthermore, depending on the mechanism, hypersensitivity reactions may be classified as either IgE-mediated or non-IgE-mediated [9,10,11,12,13]. Several studies have indicated a higher incidence of hypersensitivity reactions with ionic ICMs (4.17–12.66%) than with non-ionic ICMs (0.69–3.13%) [8,14,15].

History of adverse reactions to ICMs, irrespective of the mechanism involved, often leads to avoidance of their administration during the following diagnostic procedures, choosing different imaging techniques, or even preference of symptomatic treatment over therapeutic interventions with ICMs. These approaches are usually pernicious, and related to worse clinical prognosis. As a consequence, allergologic work-up and choosing a safe alternative ICM, or premedication are currently regarded as the best strategies to cope with the problem.

The aim of this study was to assess the negative predictive value of skin tests and the complete allergologic work-up with iodinated contrast media in real life settings.

## 2. Materials and Methods

### 2.1. Study Group

A total of 210 patients aged 31–86 (average age: 64 years) were retrospectively recruited from the group hospitalised between 2018 and 2025 in the Department of Allergology, Medical University of Gdansk, Poland. The inclusion criteria were history of immediate hypersensitivity reactions (up to 60 min after administration) to ICMs, and presence of clinical problems requiring imaging with ICM in the near future. We excluded patients with a history assessed as untypical for hypersensitivity reactions, delayed reactions as well as conditions that could affect the safety of allergologic procedures (current infection, pregnancy, respiratory or cardiac insufficiency, GFR < 60 mL/min./1.73 m^3^, etc.).

Exclusion and inclusion criteria are presented in Table 2, and the characteristics of the study group are presented in Table 3.

### 2.2. Methods

Medications that could affect the results of the tests as well as metformin were withdrawn according to general guidelines [16,17]. Allergologic work-up was performed according to current standards and recommendations for drug allergy diagnosis. Stepwise protocol of indicating a safe iodinated contrast medium included: skin prick test (SPT), intracutaneous tests (ICT), and an intravenous drug provocation test (DPT). Skin tests were performed with one or more of the following medications: iohexol, iodixanol, iomeprol, iopromide, ioversol. Culprit ICM was not tested. Despite the retrospective recruitment of study participants, all diagnostic procedures were done according to the same protocol routinely used in our clinic.

SPTs were performed in accordance with current guidelines [18]. The positive control was a prick test with histamine, and the negative control with saline. Following the control tests, SPT with undiluted ICM was performed. The SPT was considered positive if a wheal of ≥3 mm in diameter was found. The next steps involved ICT with diluted (1:10) and undiluted ICM. It was assessed as positive in the case of an increase in wheal diameter of ≥3 mm in comparison with the primary wheal diameter, and presence of erythema. If one of the steps yielded a positive reaction, the ICM was excluded from further tests.

Skin tests were followed by intravenous, placebo-controlled, single-blinded challenge tests with placebo and the following doses of ICM: 0.1 mL diluted 1:100; 0.1 mL diluted 1:10; undiluted: 0.1 mL; 1 mL; 5 mL; 20 mL. As the provocation test was a single-blinded trial, the staff were aware of the drug administered all the time. The escalation of doses was ceased in a case of:

- informed consent withdrawal;
- objective symptoms of hypersensitivity reaction (e.g., skin rash, fall in blood pressure, wheezing, etc.);
- poor compliance.

All patients were observed in hospital settings for at least 24 h after last dose administration.

DPT was assessed as positive in cases of objective symptoms of a hypersensitivity reaction. Mild reactions (urticaria, angioedema) were treated with glucocorticoids and/or antihistamines. In severe reactions (anaphylaxis) epinephrine was administered. After at least 24 h symptom-free, another substance was tested.

After the successful determination of safe ICM, the study participants were given written information regarding results of allergologic work-up, and a recommendation on using the ICM in the future. Radiologists were provided with this message prior to the planned procedures.

At least 6 months after allergologic work-up with successful selection of safe ICM a telephone follow-up visit was scheduled. Study participants were asked questions regarding ICM administration, their tolerance, and adverse events upon exposure (Table 4).

As part of the statistical analysis we compared the positivity of allergologic work-up between patients tested early (≤6 months, 6–12 months after reaction) and late (>1 year); we sought associations with the characteristics of the reaction to the ICM, concomitant disease, ICM used, age, and gender. Finally, the negative predictive value (NPV) was calculated. It was defined as a proportion of true negative results to all negative results (true negatives + false negatives). The NPV of skin tests was calculated in relation to the drug provocation test. In the next step the NPV of the whole work-up was assessed during a telephone follow-up call based on real-life ICM exposure.

For statistical analysis, Statistica software version 13 (TIBCO Software Inc., Santa Clara, California, USA) was applied. The normality of data distribution was assessed by the Shapiro–Wilk test. Normally distributed data were compared between two subgroups with the use of Student’s *t*-test. Otherwise, Mann–Whitney U-test was applied. Relations between categorical variables were tested with chi-squared test.

The study was conducted in accordance with the Declaration of Helsinki. Informed consent was taken before allergologic work-up. The protocol of the study was approved by the Independent Bioethics Committee, Medical University of Gdańsk, Poland (KB/627). The data reporting adhered to STROBE guidelines on observational studies (The Strengthening the Reporting of Observational Studies in Epidemiology) [19].

## 3. Results

A total of 210 patients were retrospectively enrolled into the study, all of whom had a history of an immediate hypersensitivity reaction to ICM. The prevailing symptoms included skin rash such as urticaria and/or angioedema (82%), shortness of breath (36%), anaphylaxis (55%); Table 3. The medical procedures that led to the reactions were mostly CT scans (54%) and coronary angiographies (21%). The interval between a reaction to the ICM and allergologic work-up varied significantly within the group (between 25 years and 3 months, mean interval—6.7 years). All of the participants either had a scheduled procedure with ICM administration, or a condition requiring monitoring with imaging tests.

Overall, 441 skin prick tests were performed, 55 of which (12%) were assessed as positive. Consequently, 383 intracutaneous tests were done with 40 (10%) positive results (Table 5). Fourteen participants (6.6%) were found to be positive in skin tests with all ICMs available at the moment of diagnostics, and this group was excluded from DPTs.

In the next step, altogether 200 DPTs were conducted. Five of them were not completed due to a response to the placebo, and the following 10 (5%) were evaluated as positive. The reactions observed were mostly mild—in eight cases they were limited to the skin (urticaria, angioedema, erythema, etc.); in two cases symptoms were systemic (urticaria and breathlessness; erythema, abdominal pain, vomiting, dizziness), and these patients required epinephrine administration and prolonged hospitalisation.

The proportion of positive diagnostics differed significantly between early- (≤1 year) and late (>1 year) diagnostics (30% vs. 15%; *p* = 0.0126). There were no differences between diagnostics performed ≤6 months and 6–12 months after the reaction, probably due to a small sample of patients tested within 6 months after the reaction. As far as the history of a reaction to the ICM is concerned, skin involvement was associated with a higher rate of positive results (*p* = 0.04). Such relations were not found for a history of anaphylaxis, breathlessness, gastrointestinal symptoms (*p* > 0.05). Concomitant diseases, patients’ age or gender, and ICM provoking the reaction were not associated with the positivity of allergologic work-up either.

**Figure 1:**
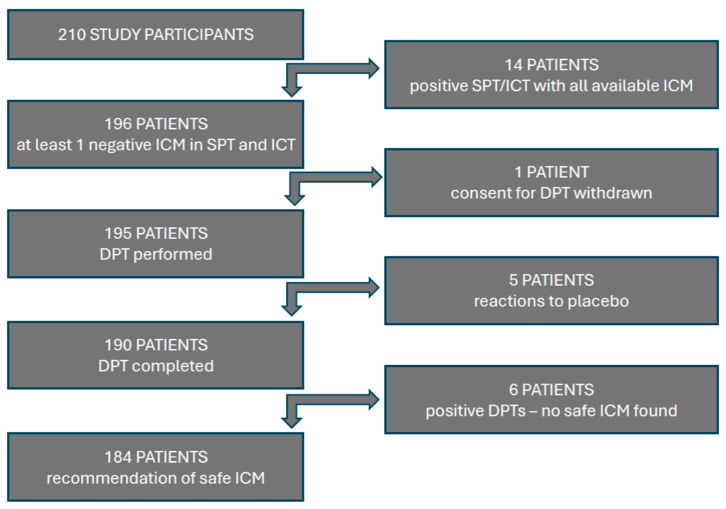
Patient flow in the study. Out of 210 patients enrolled, in 184 (87%) it was possible to recommend a safe ICM based on negative skin tests and intravenous drug provocation test. SPT—skin prick test. ICT—intracutaneous test. DPT—intravenous drug provocation test.

Thus, the negative predictive value of skin tests in reference to DPTs in hospital settings was 95%. As a matter of fact, in the vast majority of participants (184 subjects; i.e., 87% of the study group) we managed to find a safe ICM for the scheduled procedure. The flow of study participants at this stage has been presented in Figure 1.

In the final step, telephone follow-up visits were scheduled to assess the tolerance of ICMs in real-life settings in 184 patients who had been assessed as tolerant on the basis of DPT. We managed to contact 152 (82%) of the patients, and 144 (78%) answered questions as listed in Table 4; 85 participants (46%) confirmed having been given an ICM. In the vast majority, i.e., 68 cases (37%) the recommended ICM was used with no subsequent hypersensitivity reaction, and 7 (4%) patients reported a reaction despite being given the ICM tolerated upon DPT.

The reactions reported were mostly mild—acute urticaria (2 cases), delayed maculopapular rash with onset on the following day (2 cases), general weakness with a headache without objective symptoms (2 cases). In patients’ accounts these symptoms mostly subsided spontaneously. Only patients with acute urticaria were given steroids i.v. and oral antihistamine.

However, one participant reported anaphylaxis. This was a case of a 47-year-old woman with a history of previous anaphylaxis after an unknown ICM was administered. The patient was negative in SPT, ICT, and DPT with iopromide. Having been given iopromide during a CT scan, she reported breathlessness, skin erythema, and weakness and was given epinephrine i.m.

Out of the study group, 59 (32%) were not given an ICM after allergologic work-up, including 30 patients who did not need imaging, 11 patients currently waiting for the procedure, 13 patients who were refused a test with the use of ICM due to staff’s safety concerns, and 3 who did not give informed consent because of anxiety related to the possibility of a hypersensitivity reaction.

The negative predictive value of allergologic work-up consisting of SPT, ICT, and DPT in real-life settings was 90% (true negatives/true negatives + false negatives: 68/75). Considering that a significant number of patients were “lost” to follow-up (no contact, refusal to respond, etc.), we also calculated possible best-case (all lost negative) and worst-case (all lost responded) scenarios. In the former NPV augmented to 94% (108/115). In the latter fell to 59% (68/115).

## 4. Discussion

Despite the introduction of non-ionic, low-osmolar agents, reactions to ICMs still occur, ranging from a mild inconvenience, such as a heat sensation, nausea or urticaria, to life-threatening anaphylaxis. The most common adverse effects of ICMs are shown in Table 6. Risk factors for these reactions include a previous individual or family history of hypersensitivity reactions to ICM, hyperthyroidism, drug allergy, other allergies, asthma, female sex, age <35 years, high body mass index, and type of ICM used [20,21,22].

The most obvious strategy to cope with hypersensitivity to ICMs is their avoidance. On the other hand, since a substantial portion of diagnostic procedures would be completely banned, such a strategy would complicate clinical decisions, or even impair treatment implementation. In some cases, the risk related to quitting diagnostics would exceed risks associated with ICM hypersensitivity. To avoid such dilemmas, the most common strategies involve either allergologic work-up with the intention to identify a safe alternative ICM for future radiological procedure, or premedication [27].

As far as premedication is concerned, there is no general consensus regarding the recommended protocol. Some studies have demonstrated that premedication with an antihistamine is associated with a reduced risk of a breakthrough reaction [20]. In another one, the authors suggest stratification of the risk, and using several protocols depending on patients’ history (e.g., low risk: antihistamine; medium risk: antihistamine + single dose of steroid; high risk: antihistamine + repeated doses of steroid) [28]. Nevertheless, the most common approach is premedication using an antihistamine and steroid combination in patients with a history of mild immediate or delayed hypersensitivity reactions [20,29]. As premedication does not prevent anaphylaxis effectively, high-risk patients are generally regarded as candidates for allergologic work-up.

In our study we enrolled patients with varied severity of immediate hypersensitivity reactions—from isolated skin reactions to anaphylaxis. The group was characterised by advanced age, predominance of women, and high prevalence of chronic diseases. A substantial part of the participants required ICM for cardiovascular interventions (due to coronary arteries’ disease), or imaging related to pulmonary pathology, oncologic check-up, etc. All these conditions correlate with age, and so does the necessity to use imaging techniques with contrast media. As drug allergy requires earlier sensitization, repeated imaging with ICM seems to be a facilitator of development of allergy. Moreover, conditions correlating with age such as cardiovascular diseases and some common therapies (beta-blockers, ACE inhibitors) are well documented factors augmenting the severity of the reaction. In contrast to our finding, the earlier publications usually indicated an even higher risk of immediate reactions in younger people [30]. The predominance of women in our group is consistent with both the established observations regarding reactions to contrast agents, and with the general epidemiology of drug allergies [30,31,32].

In the majority of patients, the history of the reaction was related to the administration of a contrast medium during CT or angiography. This can be considered consistent with the literature, because these are the most common procedures associated with the intravenous use of ICMs [4,7]. A considerable proportion of our patients were unable to identify the contrast agent responsible for their reaction which is a significant, albeit a typical, limitation of studies based on patients’ history. This is understandable, as patients rarely remember names of occasionally administered medications, especially if many years have passed since the incident, and medical records were incomplete or have been lost.

A significant limitation of the present study is its single-centre design. All participants were recruited at a tertiary allergy centre. Patients referred for allergologic assessment may represent a highly selected population, and may not reflect the general population of individuals experiencing adverse reactions to ICMs. The results of the study may be limited in terms of their generalisability to all patients who have been exposed to iodinated contrast media. Nevertheless, the diagnostic process for suspected ICM hypersensitivity is conventionally conducted in specialised allergy centres, which necessitates a certain degree of patient selection.

A further limitation of our analysis was the wide variation in the time interval between the reaction and allergy testing. In particular, the subgroup of patients diagnosed early, i.e., within 6 months after the reaction, was small and could have impeded some comparisons. The literature emphasises that the interval has a significant influence on the diagnostic value of the tests, as the sensitivity of skin tests following the administration of iodinated contrast media is highest in the first few months after the reaction, and may decrease over time [33]. A multi-centre trial has demonstrated that skin prick testing within six months of the latest drug reaction results in a more sensitive test [34]. Our study has confirmed this phenomenon. Patients diagnosed within 1 year after the reaction to ICM were significantly more often positive in allergologic work-up compared to late-diagnostics subgroup. What is important, we could find this relation despite the fact that we did not test the culprit drug.

A similar phenomenon, whereby, over time, the allergy may gradually subside and SPTs may yield negative results, has also been described in relation to other medicines, particularly beta-lactam antibiotics. Studies on penicillin have shown that positive test results may disappear after years of avoiding the drug, and similar observations have also been reported for cephalosporins, where after several years, more than half of previously sensitised patients no longer tested positive [35,36,37].

Recruitment of patients with a history of immediate reactions justified the use of skin tests as diagnostic methods, as SPTs and ICTs are the primary tool for assessing suspected IgE-mediated reactions. In our study, the percentage of patients with at least one positive skin prick test (12%) is comparable to other studies (10.2–20.25%) [38,39,40]. However, such comparisons should be interpreted with caution because of major differences in the study populations, and diagnostic strategies. The primary aim of our work-up was to find a safe, alternative ICM for planned medical procedures instead of confirming an allergy. Thus, we avoided testing culprit drugs. Schrijvers et al. evaluated a large and heterogeneous cohort of 597 patients with suspected immediate, non-immediate, or undetermined hypersensitivity to iodinated contrast media finding positive skin tests in 13% of cases [40]. Voltolini et al., on the other hand, studied retrospectively 407 patients with a reported ICM hypersensitivity reaction from nine Italian allergy centres [39]. Allergy was confirmed by means of skin tests in 20% of the study group. The highest rates of positive results were recorded with iomeprol and iopromide. In the study of Meucci et al., a total of 98 patients were recruited, and no positive skin prick tests were reported. However, unlike our study, both Voltolini et al. and Meucci et al. performed the DPTs by also using the culprit ICM [38,39].

The low sensitivity of SPTs with ICM in some studies may be due to the fact that a significant proportion of these reactions do not follow an IgE-mediated mechanism [41,42,43]. This is indirectly confirmed by studies in which some patients, despite negative skin tests, produced positive results in intravenous drug provocation tests, indicating the presence of false-negative results and the limited sensitivity of SPTs [44]. Another explanation for the low rate of positive skin prick tests may be caused by the unawareness of the exact ICM involved in the hypersensitivity reaction by the clinical staff administering the ICM.

In the next step, 383 intracutaneous tests were done with 40 (10%) positive results. Our observations regarding intracutaneous tests are consistent with the existing literature, which indicates that ICTs have greater diagnostic value than SPTs in patients with suspected hypersensitivity to ICM. In the study of Meucci et al., nearly 10% of patients were positive in the ICT despite there being no positive SPT [38]. In the study by Kvedariene et al., 10 out of 44 patients tested positive, with only one having a positive SPT, and as many as seven having a positive immediate ICT, which clearly demonstrates the superiority of intracutaneous tests in this patient group [33]. It should be emphasised, however, that although intracutaneous tests have a greater diagnostic value than SPTs, they also have limitations. Their sensitivity is incomplete, and their values, like in SPTs, may depend both on the severity of the reaction experienced, and on the time elapsed between the incident and diagnosis [33,34]. Which is why a negative ICT result does not definitively rule out hypersensitivity to ICM. This is the reason why DPT is crucial.

Patients who tested positive to all available ICMs were labelled as allergic, and work-up was ceased. The priority of our procedure was to determine a safe ICM instead of verifying positive skin tests. As a result, we do not know the number of falsely positive SPTs and ICTs. The missing data could have an impact on the calculated diagnostic performance. However, considering a small number of patients with positive skin tests, this impact was not substantial.

Intravenous drug provocation tests using iodinated contrast media constituted the final stage of diagnosis in our study, and were performed on patients with negative skin test results, in accordance with the approach recommended in the literature [45]. We conducted these tests in a blinded manner, which reduced the risk of subjective over-interpretation of symptoms, and the cumulative dose was selected to correspond to the range commonly encountered during coronary angiographic procedures. The aim of this protocol was to replicate the conditions of actual clinical exposure whilst ensuring patient safety.

Altogether 200 DPTs were conducted. Five patients responded to the placebo and DPT was discontinued. Ten DPTs (5%) were evaluated as positive. Thus, the negative predictive value of skin tests in reference to DPTs in hospital settings was 95%. This is comparable to previous studies. Meucci demonstrated an NPV of 96% [38]. In a large cohort reported by Schrijvers et al. NPV of skin tests was 93.1% [40]. Another study demonstrated that in patients with a history of immediate hypersensitivity reactions to ICMs, the NPV of skin tests in relation to DPTs with low dose was 80% [44].

The final part of the study was a telephone follow-up call to patients after the diagnostic procedures had been completed. During the call, participants were asked questions regarding further exposure to contrast agents, and the occurrence of subsequent hypersensitivity reactions (Table 4). Out of the patients whom we have contacted, seventy-five patients confirmed that they were administered a recommended ICM (40%). Seven of those patients reported adverse effects (anaphylaxis—1 case; urticaria—2, delayed skin rash—2 cases). Unfortunately, thirteen patients were denied ICM administration by radiology staff, which confirms that radiologists still do not fully rely on diagnoses made by allergists. Thus, in real-life settings the negative predictive value of allergologic work-up consisting of SPTs, ICTs, and DPTs was 90%.

However, it should be emphasised that the data obtained were self-reported, meaning that it depended on the patients’ recollection, and subjective assessment of symptoms as well as their willingness to provide accurate information, and no objective verification such as radiology records, hospital records, or contrast administration documentation had been performed. Consequently, recall bias, misclassification bias, and reporting bias cannot be excluded. This constitutes a limitation of the study method. Nevertheless, the telephone follow-up call provided valuable data on the practical significance of diagnosis, and the long-term outcomes of patients irrespective of their age, residence and health literacy.

Real-life studies are important, because drug hypersensitivity reactions do not depend solely on the contrast agent itself [38]. They may be influenced by several variables like patient’s health status, concomitant medication, co-factors (NSAIDs, infection, physical exercise, etc.), dose, and the administration method of the drug [46,47,48]. For this reason, real-life studies, despite their limitations, provide a more accurate representation of the actual clinical situation.

Finally, it should be acknowledged that repeated exposure during the diagnostic process may theoretically lead to sensitisation or re-sensitisation. Although there is a lack of direct evidence for such a mechanism in the case of iodinated contrast media, re-sensitisation has been well documented in cohorts with beta-lactam allergy. In the study of Hershkovich et al. repeated skin tests and DPTs with penicillin showed that 2% had positive skin tests, and one case of maculopapular rash despite initial negative results [49]. In another study re-sensitisation with beta-lactams in second skin tests was demonstrated in 10.5% of the population that was found negative in DPTs [50,51]. Moreover, repeated therapeutic exposure to platinum-based chemotherapeutics has been associated with evolving sensitisation, including the conversion of previously negative skin tests to positive ones [52,53]. Seeking such similarities is somewhat speculative, and any comparison with other drug groups should be interpreted with caution. Although these observations cannot be directly extrapolated to iodinated contrast media, they suggest that repeated exposure may be relevant in drug hypersensitivity, and should be considered as a potential area for further research.

## 5. Conclusions

To conclude, the results of our study suggest that comprehensive allergy testing can be a valuable tool in the assessment of patients with a history of hypersensitivity to iodinated contrast media, and may assist in the planning of subsequent diagnostic procedures requiring contrast administration.

## Figures and Tables

**Table 1:** Main classes of ICMs currently used in clinical practice [4,5,6].

Class	Structure	Osmolality	Examples	Current Use
High-osmolar ionic monomers	Ionic monomer	5–8× plasma osmolality	Diatrizoate, Iothalamate, Metrizoate	Rarely used
Low-osmolar non-ionic monomers	Non-ionic monomer	2–3× plasma osmolality	Iohexol, Iopromide, Iomeprol, Ioversol	Widely used
Low-osmolar ionic dimers	Ionic dimer	2–3× plasma osmolality	Ioxaglate	Rarely used
Iso-osmolar non-ionic dimers	Non-ionic dimer	Similar to plasma osmolality	Iodixanol	Widely used

**Table 2:** Exclusion and inclusion criteria.

Exclusion Criteria	Inclusion Criteria
current infection	history of immediate allergic reaction after ICM (onset within 1 h after administration)
pregnancy	
current therapy with an immunosuppressant	symptoms typical for hypersensitivity reaction: anaphylaxis, urticaria, angioedema, breathlessness etc.
current anti-histamine use	
history of vasovagal reaction or untypical symptoms (anxiety, isolated dizziness, nausea, skin burning or sweating)	
delayed hypersensitivity reactions	need for usage of ICM (CT, angiography, etc.) in the near future
current exacerbation of any chronic disease (asthma, COPD, unstable angina, etc.)	
recent (30 days) cardiovascular event—myocardial infarction, stroke etc.	
uncontrolled hyperthyroidism	
renal function impairment (GFR < 60 mL/min./1.73 m^3^)	

**Table 3:** Characteristics of the study population. ICM—iodinated contrast medium.

Age	31–86 Years (Mean: 64.7 ± 12.01)
Gender	Female 144 (68%) Male 66 (32%)
Symptoms reported after past ICM administration	Urticaria/angioedema 172 (82%) Nausea/vomiting 30 (14%) Dyspnoea/cough/wheezing 76 (36%) Loss of consciousness 57 (27%) Anaphylaxis 115 (55%)
Procedure related to ICM administration	Computed tomography 113 (54%) Coronary angiography 45 (21%) Urography 18 (8.5%) Other/no data 34 (16.5%)
ICM administered	iohexol 26 (12.4%) iodixanol 7 (3.3%) iomeprol 28 (13.3%) iopromide 19 (9%) ioversol 7 (3.3%) uropolinum 18 (8.6%) other/unknown 105 (50%)
Interval between reaction and allergologic work-up	0.25–25 years (mean: 6.7 ± 6.6) Early diagnostics (≤1 year) 62 (29%) Late diagnostics (>1 year) 148 (71%)
Concomitant diseases	Hypertension 118 (56%) Diabetes 48 (23%) Coronary artery disease 66 (31%) Dyslipidaemia 60 (28%) Asthma 26 (12%) Malignancy 19 (9%)

**Table 4:** Results of telephone follow-up after successful allergologic work-up with selection of a safe iodinated contrast medium (ICM).

Have You Been Given Iodinated Contrast Media After Being Tested in the Department of Allergology?		No. Patients (%)
NO	(1) There was no need to use the ICM.	30 (16.3%)
	(2) The procedure has been performed, but without the ICM due to medical staff’s safety concerns.	13 (7%)
	(3) The procedure has been performed, but without the ICM due to my safety concerns.	3 (1.6%)
	(4) The procedure has been performed, but without the ICM due to another reason.	2 (1%)
	(5) The procedure has not been performed yet, but it is scheduled.	11 (6%)
YES	(6) The procedure has been performed with the recommended ICM—no allergic reaction.	68 (37%)
	(7) The procedure has been performed with an other than recommended ICM—no allergic reaction.	4 (2%)
	(8) The procedure has been performed with an unknown ICM—no allergic reaction.	6 (3%)
	(9) The procedure has been performed with the recommended ICM—there was an allergic reaction.	7 (4%)
	(10) The procedure has been performed with an other than recommended ICM—there was an allergic reaction.	0 (0%)
	(11) The procedure has been performed with an unknown ICM—there was an allergic reaction.	0 (0%)
(12) I do not know/I cannot remember		3 (1.6%)
(13) Refusal to respond on follow-up visit		5 (2.7%)
(14) No contact		32 (17.4%)

**Table 5:** Results of skin prick tests (SPTs), intracutaneous tests (ICTs) and intravenous drug provocation tests (DPTs) with iodinated contrast media.

ICM Tested	Number (Percentage) of Positive Results		
	SPT	ICT	DPT
iomeprol	12/112 (11%)	6/104 (6%)	3/59 (5%)
iopromide	20/72 (28%)	10/52 (19%)	2/29 (7%)
iohexol	8/72 (11%)	8/64 (12%)	1/19 (5%)
iodixanol	9/131 (7%)	10/113 (9%)	4/76 (5%)
ioversol	6/54 (11%)	6/50 (12%)	0/17 (0%)

**Table 6:** Overview of adverse reactions associated with ICMs, populations with increased risk, and prevention strategies.

Adverse Effect Category	Clinical Manifestations	Patients with Increased Risk	Risk Reduction Strategies	References
Hypersensitivity reactions (immediate)	Urticaria, angioedema, vomiting, abdominal pain, diarrhoea, dyspnoea, bronchospasm, drop in blood pressure, anaphylaxis.	Previous reaction to ICM, asthma, atopy, drug allergy, mast cell disorders.	Allergy work-up, selection of an alternative ICM, premedication.	Chiu et al., 2022 [15]
Hypersensitivity reactions (non-immediate)	Maculopapular exanthema, Stevens–Johnson syndrome, toxic epidermal necrolysis, delayed urticaria and angioedema.	Previous delayed hypersensitivity reaction to ICM.	Identification and avoidance of culprit ICM.	Tsu-Man Chiu et al., 2022 [15]
Nephrotoxicity	Post-contrast acute kidney injury, increased serum creatinine, decreased GFR.	Elderly patients, patients with basic renal insufficiency, diabetes, dehydration, heart failure.	Adequate hydration, minimization of contrast dose, use of low- or iso- osmolar ICM, dilution of ICM, acceleration of excretion, reduction in retention time of contrast agents.	Li et al., 2024 [23]
Cardiovascular	Bradycardia, tachycardia, arrhythmias, blood pressure changes.	Coronary artery disease, heart failure, arrhythmias.	Hemodynamic monitoring, optimisation of cardiovascular status, appropriate patient selection.	Bottinor et al., 2013 [24]
Immunotoxicity	Mast cell and basophil activation, mediator release, IgE-mediated and non-IgE-mediated reactions, T-cell mediated delayed reactions.	Patients predisposed to hypersensitivity reactions.	Allergy work-up, selection of a safe alternative ICM, careful monitoring during administration.	Tsu-Man Chiu et al., 2022 [15]
Thyroid dysfunction	Iodine-induced hyperthyroidism, hypothyroidism, transient thyroid dysfunction.	Graves’ disease, predialysis chronic kidney disease and end-stage renal disease.	Thyroid function assessment, cautious use of ICM in patients with known thyroid disease, prophylactic antithyroid drug therapy in selected patients.	Lee et al., 2014 [25]
Neurotoxicity	Headache, confusion, seizures, aphasia, cortical blindness, contrast-induced encephalopaty.	Patients with prior stroke, renal dysfunction, heart failure.	Decreasing procedure time, contrast dose, vasospasm prevention with nimodipine, hydration, ophthalmologic examination if vision deteriorates.	Vazquez et al., 2022 [26]

## Data Availability

Study database is available on demand.

## Abbreviations

CT	computed tomography
DPT	intravenous drug provocation test
ICM	iodinated contrast media
ICT	intracutaneous tests
SPT	skin prick tests
